# Traumatic Brain Injury-related voiding dysfunction in mice is caused by damage to rostral pathways, altering inputs to the reflex pathways

**DOI:** 10.1038/s41598-019-45234-8

**Published:** 2019-06-14

**Authors:** Onder Albayram, Bryce MacIver, John Mathai, Anne Verstegen, Sean Baxley, Chenxi Qiu, Carter Bell, Barbara J. Caldarone, Xiao Zhen Zhou, Kun Ping Lu, Mark Zeidel

**Affiliations:** 1 Division of Cardiology, Department of Medicine, Medical University of South Carolina, Charleston, SC 29425 USA; 2 Hematology and Oncology Division, Department of Medicine, Beth Israel Deaconess Medical Center, Harvard Medical School, Boston, MA 02215 USA; 3 Division of Translational Therapeutics, Department of Medicine, Beth Israel Deaconess Medical Center, Harvard Medical School, Boston, MA 02215 USA; 4 Cancer Research Institute, Beth Israel Deaconess Medical Center, Harvard Medical School, Boston, MA 02215 USA; 5 Division of Nephrology, Department of Medicine Beth Israel Deaconess Medical Center, Harvard Medical School, Boston, MA 02215 USA; 6 Broad Institute of Harvard University and Massachusetts Institute of Technology, Cambridge, MA 02142 USA; 7 NeuroBehavior Laboratory, Harvard NeuroDiscovery Center, Harvard Medical School, Boston, MA 02115 USA

**Keywords:** Brain injuries, Diseases of the nervous system

## Abstract

Brain degeneration, including that caused by traumatic brain injury (TBI) often leads to severe bladder dysfunction, including incontinence and lower urinary tract symptoms; with the causes remaining unknown. Male C57BL/6J mice underwent repetitive moderate brain injury (rmdTBI) or sham injury, then mice received either *cis* P-tau monoclonal antibody (*cis* mAb), which prevents brain degeneration in TBI mice, or control (IgG). Void spot assays revealed age-dependent incontinence in IgG controls 8 months after injury, while *cis* mAb treated or sham mice showed no dysfunction. No obvious bladder pathology occurred in any group. Urodynamic cystometry in conscious mice revealed overactive bladder, reduced maximal voiding pressures and incontinence in IgG control, but not sham or *cis* mAb treated mice. Hyperphosphorylated tau deposition and neural tangle-like pathology occurred in cortical and hippocampal regions only of IgG control mice accompanied with post-traumatic neuroinflammation and was not seen in midbrain and hindbrain regions associated with bladder filling and voiding reflex arcs. In this model of brain degeneration bladder dysfunction results from rostral, and not hindbrain damage, indicating that rostral brain inputs are required for normal bladder functioning. Detailed analysis of the functioning of neural circuits controlling bladder function in TBI should lead to insights into how brain degeneration leads to bladder dysfunction, as well as novel strategies to treat these disorders.

## Introduction

Overactive bladder, incontinence and other bladder symptoms often occur in patients with neurodegenerative disease or brain injury, and can lead to significant debility, striking loss of self-esteem, depression, and the need for long term institutionalization^1–4^. At present the mechanisms by which neurodegenerative diseases lead to severe bladder dysfunction remain entirely unclear.

Although severe or/and moderate injuries most frequently sustained from single events falls, assaults, or roadside traffic accidents, which can lead to early death and profound disability^5,6^, military and sport-related injuries have received increasing attention in the past decade^7–10^. Military-based brain injuries result in several ways; directly from the percussive wave, from blast induced projectile impacts, or from blast induced falls^9–11^. Traumatic brain injury (TBI) represents a leading cause of death and disability among people under the age of 45 in the United States^12^. For survivors, the sequelae of brain injuries are substantial and debilitating, requiring 5–10 years of intensive therapy, and resulting in permanent disability^12^. Along with adverse cognitive, emotional and functional outcomes after brain injuries, TBI is one of the strongest environmental risk factors for chronic traumatic encephalopathy (CTE)^13,14^, it is estimated that 57 million people worldwide are living with CTE. In many respects CTE resulting from TBI resembles other forms of degenerative brain injury, including Alzheimer’s disease^15,16^, Lewy Body Dementia^17^, and Parkinson’s disease^18,19^. Interestingly, there are compelling evidences support that CTE seen in veterans with blast induced TBI is similar to that found in athletes^20–24^. Our previous report also showed similar tau pathologies in diffuse axons in brain sections from 8 athletes, and 8 athletes plus military service, who were diagnosed post-mortem with CTE, along with 8 age-matched normal controls^25,26^.

A pathological signature of CTE brains is neurofibrillary tangles made of hyperphosphorylated tau with an indistinguishable pattern^12,13,27^. Tau-pathology spreads in the brain^28–30^ and can be ameliorated by certain tau antibodies^31–35^. However, since neurofibrillary tangles were not obvious acutely and sub-acutely after TBI^12,26–28^, the role of tau-pathology in TBI short and long-term consequences is unknown.

A prolyl isomerase, Pin1, isomerizes the phosphorylated Thr231-Pro (pT231-tau) motif in tau from the *cis* to the *trans* conformation^36–38^. It has been recently identified that *cis* isomer of phosphorylated pT231 motif of tau (*cis* pT231-tau, or *cis* P-tau) is an early driver of neurodegeneration prior to the accumulation of tau tangles in AD and CTE and after TBI^39^. Notably, *cis* P-tau can be effectively eliminated by *cis* pT231-tau monoclonal antibody (*cis* pT231-tau mAb, or *cis* mAb), which is strikingly potent in treating the molecular, neurologic and behavior defects of mice suffering various types of TBI^26,40,41^.

Neural control of bladder filling and voiding requires coordinate function of several components of the sacral spinal cord, various brainstem nuclei, and the adaptive control of more rostral brain regions^42^. As the bladder fills, stretch receptors in urothelium and detrusor signal to the sacral cord, activating afferent neurons which project to the periaqueductal gray (PAG) that found in the midbrain^43,44^. Initially, the activation of these neurons stimulates inhibitory GABA-ergic neurons which project to the glutamatergic neurons of the Pontine Micturition Center (PMC; Barrington’s nucleus) found in brainstem and suppress the activation of these neurons^45–47^. As the bladder reaches capacity, the intensity of impulses reaching the PAG increases, leading to shut off of the PAG GABAergic neurons and activation of PAG glutamatergic neurons projecting to the PMC. The resulting activation of PMC glutamatergic neurons causes detrusor contraction and voiding. At several points in this sequence input from more rostral domains, such as the prefrontal cortex, the lateral hypothalamus, the amygdala and other sites may suppress or augment the activities of the brainstem neurons involved^45^.

Because TBI is itself a common cause of neural disease and because CTE resembles other forms of neurodegeneration, animal models of single severe TBI and repetitive mild/or moderate TBI have been developed, and these affects a range behavioral function including mood regulation, memory and attention^48,49^. TBI-related urinary incontinence, which has been well described in human brain trauma^50–56^, is directly translatable to our preclinical closed-head TBI models where it has been found that injured mice had defective spontaneous voiding pattern. Several studies using urodynamics on TBI patients have reported up to 85% of patients with bladder dysfunction^57,58^. Moreover, this dysfunction can persist longer term as reported by Khan *et al*. (2016) where 34% of the group studied had combined bladder and bowel issues up to 5 years after a brain injury^59,60^. Bladder dysfunction that remains after rehabilitation contributes to a higher risk factor for a patient to be re-institutionalized^61^. We have no information as to how TBI leads to bladder impairment.

A recent study showed that both severe and repetitive mild brain injury in C57BL/6J male mice caused an incontinence phenotype and in both cases this phenotype was rescued by the application of *cis* mAb^26^. Here we extend these previous studies, and show, using urodynamic measurements and real-time thermal imaging of voiding pattern in conscious mice and that mice receiving repetitive moderate (rmd)TBI but not sham control develops overactive bladder phenotypes and incontinence, without any obvious bladder pathology. Treatment of rmdTBI mice with *cis* mAb prevented the bladder disturbances. Histology on brain slices reveals robust tau hyper phosphorylation a long with post-traumatic neuroinflammation in the cortex but not in the mid and hindbrain regions where the PAG and PMC are found. These results indicate that TBI and possibly other forms of brain degeneration disrupt normal bladder function likely by altering profoundly the normal neural traffic to PMC and PAG bladder control centers, and not by direct damage to the control centers themselves. We believe that this model of brain injury will be applicable to elucidating mechanisms of bladder dysfunction for many forms of brain degeneration.

## Results

### Age-dependent urinary incontinence occurred time after brain injury in mice without any urinary bladder pathology and recovered by specific tau antibody

**Figure 1:**
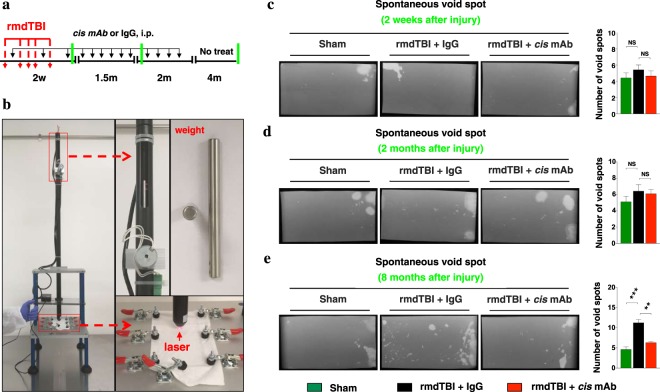
Repetitive moderate TBI changes urinary pattern in chronic, but not in acute phase, which is fully rescued by antibody therapy. (**a**) Two-month-old WT mice were subjected to sham-operation or rmdTBI but were also treated with either *cis* mAb or IgG isotype control for up to 4-months after the last injury with (**b**) newly designed TBI machine. Experimental treatment setup (Red arrows, rmdTBI, Black arrows, antibody injection; green lines, spontaneous voiding assay). Changes in urinary incontinence and voiding pattern were analyzed using the spontaneous voiding assay at (**c**) 2-weeks, (**d**) 2-months, and (**e**) 8-months after the last injury. NS: not significant. N = 4–5. The data were presented as means ± SEM. The p values were calculated using one-way ANOVA with post-hoc Tukey test. ***p < 0.001.

Mice undergoing head trauma received five moderate injuries in 7 days (54-gram weight, 87 cm drop height, (Fig. 1a,b), repetitive moderate or rmdTBI and were treated with multiple doses of rmdTBI + *cis* mAb (*cis* mAb) or rmdTBI + IgG (IgG) control during the 2 weeks of repeated injury, and over the 4 months thereafter, followed by 4 months of washout without treatment, as described in Methods (Fig. 1a). Figure 1c–e shows characteristic void spot patterns and quantifies, in all mice, the numbers of urinary spots, which corresponds roughly to total volume of urine voided (Fig. S1). In all mice, void spot assays, which assess spontaneous conscious voiding, revealed normal voiding immediately after repetitive moderate head trauma (at 2 weeks) and for 2 months thereafter (Fig. 1c,d). Placebo IgG control led to an incontinence phenotype, as measured by void spot assays, at 8 months after injury (Fig. 1e). Voiding was normal in sham TBI mice at all time points. Treatment with *cis* mAb prevented the development of urinary incontinence as detected by spontaneous voiding assay (Fig. 1e). Statistical analysis revealed similar voiding volumes among the groups at all times post trauma (Supp. Fig. 1). By contrast, IgG treated mice exhibited statistically increased numbers of urinary spots at 8 months after injury, as compared with sham and *cis* mAb treated mice. Socially dominant male mice promiscuously urinate small volumes, dispersing urine throughout the entire enclosure, whereas subordinate males urinate in a spatially smaller restricted manner^62^. Spontaneous voiding assay showed that there was oftentimes one dominant male (alpha male) in each cage of grouped housed mice. Such a phenotype could result from abnormalities in the bladder itself or in its control by brainstem centers.

**Figure 2:**
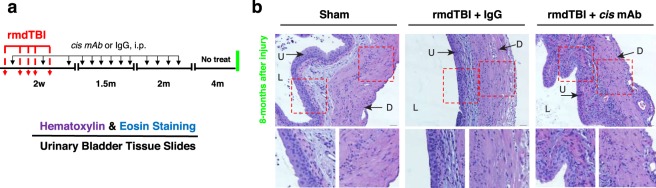
There is no obvious bladder pathology in 8-months after repetitive moderate TBI. (**a**) Two-month-old WT mice were subjected to sham-operation or rmdTBI but were also treated with either *cis* mAb or IgG isotype control. Experimental treatment setup (Red arrows, rmdTBI, Black arrows, antibody/IgG injection; green lines, bladder histopathology). (**b**) Bladder sections of sham, rmdTBI + IgG, and rmdTBI + *cis* mAb mice were subjected to hematoxylin & eosin staining at 8 months after injury. Inset images are high magnifications of representative areas with corresponding images above. L = Lumen, U = Urothelium, D = Detrusor. Scale bar, 20 μm.

Given the clear abnormalities in spontaneous voiding function in IgG mice, we examined whether the rmdTBI protocol led to long-term changes in bladder structure. Eight-months after the last injury (Fig. 2a), bladders were fixed in formaldehyde, paraffin embedded, sectioned and stained with hematoxylin and eosin. Despite the marked change in function in IgG mice, we could discern no change in morphology among the 3 groups (Fig. 2b).

### Conscious cystometry indicates overactive bladder phenotype in rmdTBI mice

We evaluated urodynamic function using a conscious cystometry (CMG) approach, which enabled us to perform multiple tests on each animal (Fig. S2). Sixteen mice were used for this part of the study with the investigators performing these experiments being blinded to the treatment the animals had received. A surgical procedure placed a catheter into the bladder dome with the catheter exteriorized at the neck and protected by a harness. This arrangement permits ongoing measurement of bladder pressure and bladder filling through the catheter without interfering with the bladder outflow path. Mice were given at least 5 d to recover from the surgery after which they received a series of conscious CMGs with saline infusion via syringe pump at 25 µl/min by being placed in a modified metabolic cage where thick blotting paper was placed over the cage’s grid (Fig. S2). Each test lasted approximately 1 h and mice were tested 3–4 times with tests occurring about the same time on different days.

Data were collected in the form of CMG pressure tracings along with an infra-red video from below (to detect each void as the warm urine soaked into the filter) and normal video from above (to record the movement of the mouse). Analysis was completed by correlating voids to the recorded cystometrogram using both the infra-red and normal video recording. Data collected were inter void interval and maximum to minimum pressure during the void. For very uniform CMGs additional data can be extracted, such as compliance^63^. However, some of these CMGs recorded quite abnormal voiding patterns and such additional measurements could not be made reliably. The blotting paper was also imaged under ultra violet light to record the overall voiding pattern at the end of the CMG. Of 16 mice subjected to CMG, 2 exhibited surgical complications of catheter placement and 14 completed the CMG protocol. Of these fourteen mice, 5 had clearly abnormal CMGs, 6 had normal CMGs and 3 were classed as uncertain. The 5 classed as abnormal were all IgG control mice, the 6 normal were 3 sham and 3 *cis* mAb mice. The 3-uncertain broke down as follows: one had a voiding pattern resembling normal mice, around 5–7 large voids during the course of the experiment, but its CMG profile was somewhat abnormal – this was a *cis* mAb mouse, one had a normal CMG pattern but somewhat increased inter void intervals, 14–16 voids in the hour – this was a sham mouse and the third exhibited variable CMG across the 3 experiments conducted – this was a *cis* mAb treated mouse.

**Figure 3:**
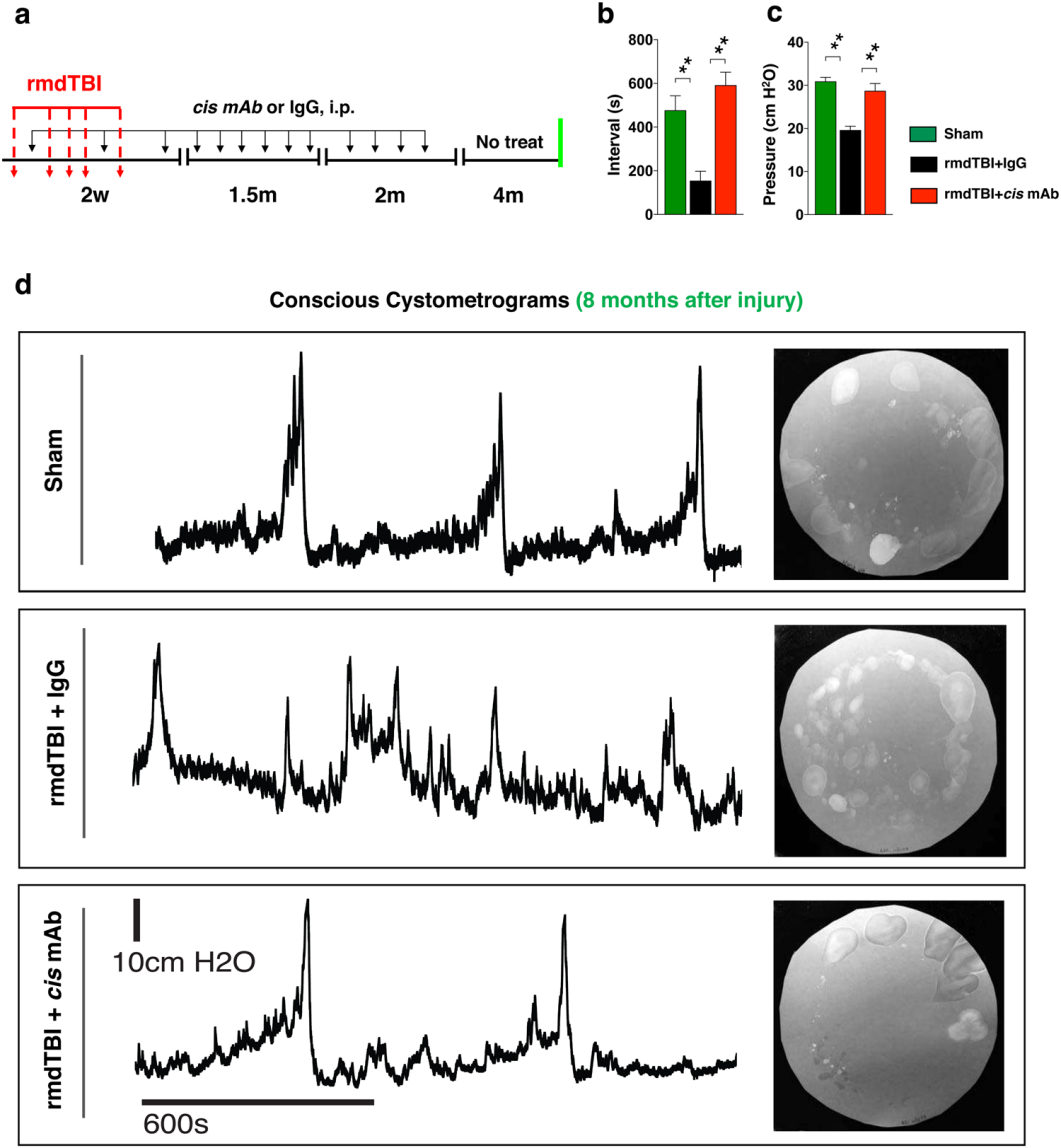
Repetitive moderate TBI changes urodynamic properties at 8-months after injury. (**a**) Two-month-old WT mice were subjected to sham-operation or rmdTBI but were also treated with either *cis* mAb or IgG isotype control. (**a**) Experimental treatment setup (Red arrows, rmdTBI, Black arrows, antibody/IgG injection; green lines, conscious cystometrograms [CMG] assay). **(b,d**) Inter void intervals are significantly shorter in TBI + IgG mice, whereas sham and TBI + *cis* mAb treated mice do not differ significantly. (**c**,**d**) Peak voiding pressures are significantly reduced in rmdTBI + IgG mice. N = 4–5. The data were presented as means ± SEM. The p values were calculated using one-way ANOVA with post-hoc Tukey test. **p < 0.01.

IgG controls exhibited significantly increased voiding frequency, with a lower inter void interval of 155 ± 43 s compared to 477 ± 66 s for sham and 592 ± 59 s for *cis* mAb treated mice, (Fig. 3a,b) (p < 0.01). The inter void interval between sham and *cis* mAb treated mice were not significantly different. Peak pressure was also significantly lower at 19.6 ± 0.9 cm H_2_O in IgG mice (Sham 30.9 ± 0.9 cm H_2_O and *cis* mAb 28.7 ± 1.7 cm H_2_O. (Fig. 3c) (p < 0.01). Again, the difference between sham and *cis* mAb treated mice was not significant (Fig. 3c). Representative CMG profiles are shown Fig. 3d. Supplemental Figures provides individual CMG profiles for each of the 14 mice (Figs S3–5). These results reveal an early voiding phenotype consistent with irritable bladder in the IgG mice.

### Thermal video assay shows the age-related changes in micturition pattern in rmdTBI mice

**Figure 4:**
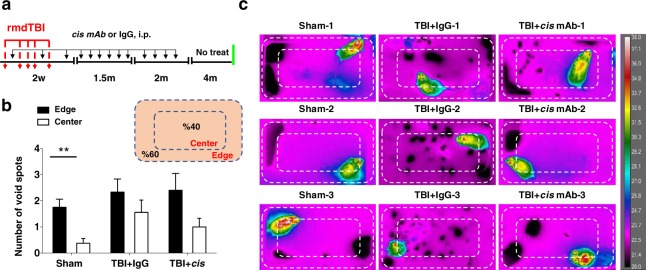
Long-term effects of repetitive moderate TBI on micturition pattern were analyzed by thermal video recording. (**a**) Two-month-old WT mice were subjected to sham-operation or rmdTBI but were also treated with either *cis* mAb or IgG isotype control. Experimental treatment setup (Red arrows, rmdTBI, Black arrows, antibody/IgG injection; green lines, thermal video recording). (**b**) Schematic representation of cage area with 40% center boundary shown by inner dotted line. Average primary voids on edge or in center as defined by voids that registered red (~35 °C) on temperature scale when micturition occurred for each group at 8-months after the last injury. (**c**) Snapshot of thermal video recording after 2 hours of 5 mice from each group. Voids become darker over time through cooling via evaporation. Dashed box represents central 40% of cage area. Scale to right is calibrated temperature in degrees Centigrade. N = 8–10 each. The data were presented as means ± SEM. The p values were calculated using two-way ANOVA with post-hoc Bonferroni test. **p < 0.01.

We evaluated the changes in micturition pattern using a thermal video assay approach, which enabled us to perform real time imaging of voiding pattern on each animal. This experiment was conducted after the 8-month void spot assay and prior to cystometry (Fig. 4a). Previous studies have shown that C57BL/6J mice prefer to void in the corners or on the edges rather than in the center^64,65^. We counted primary voids, defined as voids sufficiently large to produce a thermal response that reached red (~35 °C) on the temperature scale (see also Supplemental Data for example Videos). At the end of the video sequence a digital snapshot image was captured over which a mask representing 40% of the cage area was placed in the center and voids inside this were counted as central (Fig. 4b). Voids that spanned the mask were assigned based on where the approximate center of the void was located. It should be noted that IgG control mice produced many small spots as also seen on the void spot assay, but in the thermal video assay these are not readily quantifiable. Statistical analysis revealed that although the number of void spots was similar in both position - Edge vs Center - (F 2, 48 = 1.988, p = 0.148) it was significantly affected by the injury (F (1, 48) = 10.08, p = 0.0026) (Fig. 4b,c). In brief, the number of void spots was not influenced by the position, but significantly influenced by injury.

### Age dependent cognitive defects in TBI mice are closely correlated with neurogenic voiding dysfunction

**Figure 5:**
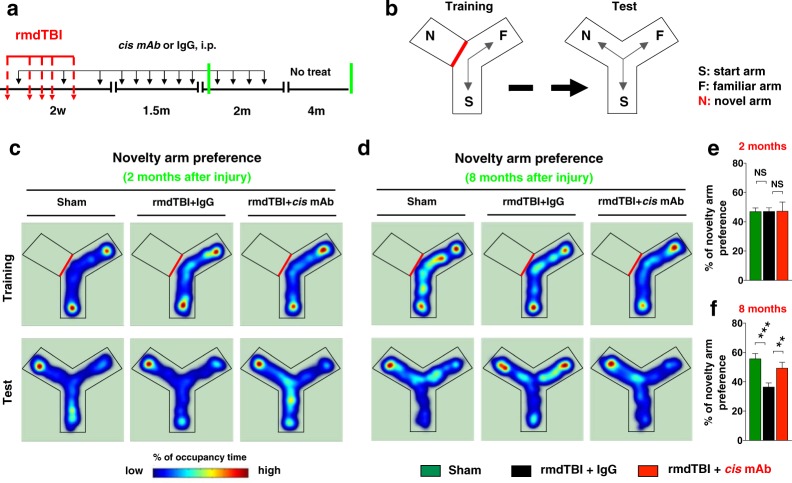
Repetitive moderate TBI induces cognitive impairment in chronic, but not in acute phase. (**a**) Two-month-old WT mice were subjected to sham-operation or rmdTBI but were also treated with either *cis* P-tau mAb or IgG isotype control for up to 2 (**c**,**e**) and 8 (**d,f**) months after the last injury. Experimental treatment setup (Red arrows, rmdTBI, Black arrows, antibody injection; green lines, cognitive tests). (**b**) The design of the standard novelty Y-maze test. During a 3-min training session (left panel), mice were allowed to explore two arms (Start and Familiar) of a 3-arm. After 1-min delay, the mice were returned to the maze for the Test session (3-min duration), during which they were now able to explore freely all three maze arms, including the previously unvisited (novel) arm. (**c**,**d**) Heat-map representing the mouse position in the experimental arena, red = more time, blue = less time. (**e,f**) The novelty arm preference was presented as means ± SEM of the percentage. The p values were calculated using one-way ANOVA with post-hoc Tukey test. **p < 0.01, ***p < 0.001.

To determine whether the rmdTBI protocol altered short- and long-term cognitive function, we performed Novelty Y-maze testing in 2 and 8 months after the last injury (Fig. 5a). Spatial learning and memory were assessed in two separate sessions, a training session and a test session. In training session, mice were pseudo-randomly assigned two arms (the “start arm” and the “left or right arm”) to which they were exposed and allowed to freely explore for 3 min. After a short interval (1 min), during the test session, the mice allowed to explore freely all three arms of the maze for 3 min (Fig. 5b). To avoid the posible carry-over effects, the same mice assigned the two arms (the “start arm” and the left or right arm”) at 2-months after the last injury in the training session of the test, and then were retested 6-months later in the same maze but in a different training arms. Two-months after the last injury (Fig. 5c,e), we did not detect any impairment preference to novel arm in all three groups. Functionally, rmdTBI did not lead to deficits in the novel location recognition at 2 months after injury. Eight-months after the injury, rmdTBI but not sham mice showed clear working memory deficits in the Novelty Y-maze test compared with sham control, a phenotype which was rescued completely by treatment with *cis* mAb, but not with IgG controls (Fig. 5d,f). We and others have observed this behavioral phenotype in TBI^1,27,49,66^, but not sham TBI mice, indicating that the rmdTBI protocol led to impairment of neurocognitive function in chronic phase. There is no obvious difference in locomotor activity or anxiety-like behavior throughout tests at 2-and 8-months after injury, which analyzed by the number of arm entries and total distance travelled in each training and test sessions at (Fig. S6a–d) 2-months, and (Fig. S6e–h) 8-months after the last injury.

### Neuropathological lesions are well correlated with voiding dysfunction at 8 months after injury

**Figure 6:**
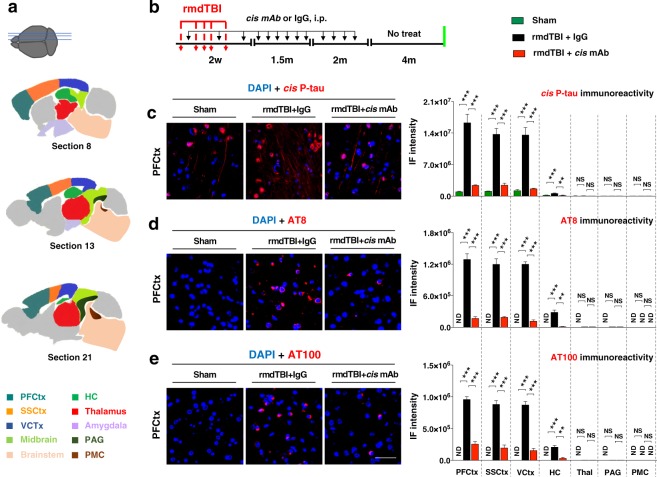
Repetitive moderate TBI induces long-term tau pathologies, but cis mAb therapy attenuates those detrimental effects of TBI. (**a**) Anatomical representation in sagittal sections with regions color-coded according to modular affiliation. Sections are numbered as in the Allen Reference Atlas; Inset shows section locations in sagittal plane. (**b**) Two-month-old WT mice were subjected to sham-operation or rmdTBI but were also treated with either *cis* mAb or IgG isotype control. Experimental treatment setup (Red arrows, rmdTBI, Black arrows, antibody/IgG injection; green lines, histological assays). Mouse sagittal brain sections were analyzed by IF for (**c**) *cis* P-tau, (**d**) AT8 and (**e**) AT100 at 8 months after the last injury. Scale bar, 20 μm. ND: not detectable; NS: not significant. PFCtx: Prefrontal cortex; SSCtx: Somatosensory cortex; VCtx: Visual cortex; HC: Hippocampus; Thal: Thalamus; PAG: Periaqueductal gray; PMC: Pontine micturition center. Brains from 4 male mice were studied for immunohistochemistry. The data were presented as means ± SEM. The p values were calculated using one-way ANOVA with post-hoc Tukey test. **p < 0.01, ***p < 0.001, ****p < 0.0001.

Because of traumatic brain injury led to cognitive, spontaneous voiding and CMG defects in IgG control mice, but not sham or *cis* mAb treated, we examined the brain of each mouse in detail, looking for evidence of degenerative changes. In particular, given the early voiding and irritable bladder phenotype, we wondered whether rmdTBI led to damage to brain sites known to provide reflex control of bladder filling and voiding, Barrington’s Nucleus (Pontine Micturition Center, PMC) as well as a major site which projects to this region, the periaqueductal gray (PAG) (Fig. 6a). In addition, we examined more rostral sites which also project to the PMC. As shown in Fig. 6b, in IgG mice at 8 months after injury, we observed robust *cis* P-tau accumulation as shown by immunofluorescence staining (IF) for *cis* P-tau (Figs 6c and S11), and IF staining with AT8 (Figs 6d and S7a) and AT100 mAbs (Figs 6e and S7b) to detect tau hyperphosphorylation, and silver staining (Fig. S8) to detect tangle-like pathology in prefrontal cortex, secondary somatosensory cortex, ventral cortex, and hippocampus, while thalamus, PAG and PMC were entirely normal. Sham and *cis* mAb treated mice exhibited little if any *cis* P-tau accumulation and the antibody treated mice exhibited little or no evidence of tau pathologies. We also ask whether reducing the deposition of hyperphosphorylated tau protein, does effect on post-traumatic neuroinflammation, which is one of the major secondary mechanism known to promote continuous tau hyperphosphorylation and accelerate disease progression^67–70^. We observed that reducing tau pathologies in cortical regions, but not in deeper brain regions, are mitigated post-traumatic neuroinflammation, as assayed by immunofluorescence (IF) staining of Iba1 to detect microglial activation (Fig. S9). We also did not observe any significant difference in neuronal density in PMC and PAG sub regions among the groups (Fig. S10).

These results are entirely consistent with prior studies on TBI mice from our group and others. It is notable that we detected no deposition of *cis* P-tau or evidence of tau pathologies in the PAG or PMC regions. Notably, changes observed soon after injury were limited to the subcortical regions and hippocampus close to the impact site, but not found in deeper brain regions, such as the midbrain and brainstem, including PAG and PMC, respectively (Figs 6 and S7–10).

## Discussion

Degenerative brain disease in humans often leads to severe bladder dysfunction, which can include overactive bladder and frank incontinence. Bladder symptoms lead to social isolation, depression and often precipitate institutionalization and accelerate functional decline. Although the disease burden of bladder dysfunction in brain degenerative disease is enormous, we know little about how damage to the brain leads to bladder dysfunction, and this lack of knowledge prevents the development of rational therapeutic approaches to alleviate these symptoms. Although investigators have developed numerous genetic models of brain degeneration, including models that feature tau or amyloid deposition, each model has limited applicability to brain degeneration in general, and it can be difficult to distinguish the idiosyncratic from the generalizable features of each model. By contrast, TBI is relatively common and leads to clear cut brain degeneration. Models incorporating rmdTBI lead to brain degeneration which models human TBI as seen in collision sports or military trauma, with well characterized loss of cognitive and motor function and, in our studies, with loss of normal bladder function^6–11,20,54,59–61^.

There are profound differences in histopathological responses even between different types of closed head injury models. Each model of injury imitates the diffuse axonal injury differently because of the diverse spectrum of mechanisms that can occur as a function of injury severity, injury location and time. There are various unique mechanisms, including repetitive mild/moderate TBI as seen in collision sports and military blasts; and single moderate/severe TBI as seen in assaults or motor vehicle accidents, cause different acute and potentially long-lasting behavioral and neuropathological features^1,5–11^.

To date, studies linking TBI and bladder function in rodents have been limited to rats, have employed a different form of TBI (fluid wave), and have resulted in urinary retention followed by incontinence, with clear-cut changes in bladder structure^71–73^. Normal bladder filling requires the processing in the brain of afferent impulses sensing bladder stretch, with suppression of detrusor firing and sphincter relaxation until a threshold filling is reached. At this point the brain (likely the ventrolateral periaqueductal gray (PAG), which receives direct sensory stretch input from sacral afferents) switches from inhibition to stimulation, causing reflex contraction of the detrusor and release of the sphincter, to permit voiding. These reflex arcs are controlled by more frontal regions which modulate their activity to the needs of the animal in the environment. Normal mice are continent and, in several instances, such as male dominant and submissive behavior, voiding is used for communication; on this basis mice can model normal and deranged bladder function^62,63,74,75^.

Modern neurobiology methods have identified neurons in Barrington’s nucleus of the Pons (the pontine micturition center (PMC) which, when fired, contract the detrusor (Glutamatergic and corticosteroid releasing hormone expressing neurons, designated PMC^CRH^ neurons and PMC^GLUT^ neurons, respectively) and release the sphincter (estrogen receptor neurons, designated PMC^EST^ neurons). Current study showed PMC^CRH^ neurons capable of driving bladder contractions with retrograde mapping showing inputs from a large number of brain regions^47^. Furthermore, a neuroanatomical study showed these CRH neurons having “unexpectedly long” dendrites strongly supporting inputs from many other brain regions^76^. Given the large number of inputs from frontal brain regions into the PMC, further experimentation using this TBI model along with current mapping techniques will enable more precise understanding of how the neuronal inputs into the PMC act to control voiding.

These neurons receive a complex array of inputs, which include inhibitory (GABA-ergic) and stimulatory (Glutamatergic) neurons from the ventrolateral PAG, other midbrain sites including the reticular nucleus and superior colliculus, as well as 10–13 other sites, including, within the hypothalamus, the lateral and paraventricular areas, medial preoptic area^76,77^, and the zona incerta. Additional afferents to these PMC neurons arise from the amygdala, the motor, somatosensory, anterior cingulate, orbito-frontal and prelimbic regions of the cortex, and the bed nuclei of the stria terminalis. Thus, in the mouse, ongoing work is mapping the circuits which control bladder filling and voiding, and which integrate bladder function with the needs of the organism^74–77^. The relevance of these findings in mice to human bladder function is underscored by functional imaging studies in humans, which have shown the equivalent regions in humans are important for processing input into voiding control.

Our results demonstrate clearly that rmdTBI leads to cognitive dysfunction and brain degeneration, and that treatment with *cis* P*-*tau antibody prevents both the post-traumatic brain degeneration and the cognitive dysfunction. Also, we have shown that rmdTBI causes incontinence, and *cis* P-tau antibody treatment prevents the incontinence phenotype. Thus, rmdTBI + IgG compared with rmdTBI + *cis* mAb represents an excellent and well controlled model by which to study the mechanisms by which brain degeneration leads to incontinence. We find that the incontinence develops over 8 months after rmdTBI, coincident with the various tau pathologies and post-traumatic neuroinflammation, and it is absent in rmdTBI mice in which the brain degeneration is prevented by *cis* mAb treatment. The incontinence phenotype is manifest on CMG as irritable bladder, with early voiding and detrusor contractions, indicating an inability of the bladder to fill properly before voiding and bladder contraction is initiated. The defect is not in the bladder itself, because the bladders from all mice were entirely normal. Therefore, the defect must be in the neural control of the bladder and may result from changes in the afferent signaling from sacral cord to PAG, the “switch” mechanism from accommodation of filling to voiding and contraction in the PAG, or premature activation of PMC^GLUT^ and PMC^EST^ neurons without an alteration in PAG activity.

Importantly, careful study of the brains of our mice reveals no structural differences or labeling for *cis* P-tau or evidence of hyperphosphorylated tau or neurodegeneration in the PMC and PAG regions, the regions responsible for reflex control of bladder filling and voiding, are entirely intact. By contrast, IgG mice, but not sham or *cis* mAb treated mice exhibited striking accumulation of *cis* P-tau and hyperphosphorylated tau epitopes in prefrontal cortex, secondary somatosensory cortex, ventral cortex, and hippocampus. On this basis, the incontinence caused by TBI-induced brain degeneration must result from derangements in the neural input to nuclei like the PAG and PMC from frontal areas that undergo degeneration as a result of TBI, and not from dysfunction of the nuclei themselves. These results open the way for detailed studies of the function of the reflex arcs (in PMC and PAG neurons) controlling bladder filling and voiding in rmdTBI mice as well as careful examination of how rmdTBI alters afferent traffic from rostral regions that degenerate, to these nuclei. Such studies may provide important information as to how brain degeneration in more frontal parts of the brain leads to disturbances in bladder function. In addition, it may be possible, in these mice, to test approaches to preventing premature bladder contraction and voiding, for example, by programmed selective stimulation of ventrolateral PAG GABA-ergic neurons, which can inhibit voiding and bladder contraction.

## Materials and Methods

### Traumatic brain injury

The mouse closed head injury model was used as previously described^78^. Briefly, male C57BL/6J mice (2–3 months old) obtained from the Jackson Laboratories (Bar Harbor, ME) were randomized to undergo injury or sham-injury. The mice were anesthetized for 45 s using 4% isoflurane in a 70:30 mixture of air: oxygen. Anesthetized mice were placed on a delicate task wiper (Kimwipe, Kimberly-Clark, Irving, TX) and positioned such that the head was placed directly under a hollow guide tube. Mouse’s tail was grasped. A 54 -gram metal bolt was used to deliver an impact to the dorsal aspect of the skull along an 87 cm height tube, resulting in a rotational acceleration of the head through the Kimwipe. For repetitive moderate TBI (rmdTBI), mice underwent 5 injuries in 7 days. Sham-injured mice underwent anesthesia but not concussive injury. All mice were recovered in room air. Anesthesia exposure for each mouse was strictly controlled to 45 s. Briefly, anesthetized young adult wild-type C57BL/6 male mice were exposed to a moderate hit or sham hit, removed from the apparatus, monitored until recovery of gross locomotor function, and then transferred to their home cage. Maximum burst pressure was compatible with 100% survival and no gross motor abnormalities were ascertained empirically. For all studies of behavioral and bladder phenotypes, as well as histology, observers were unaware of which experimental group the mice were in while determining the results of measurements.

### Antibody treatment of mice

C57BL6/J male mice (2−3 months old) undergoing rmdTBI were randomized to treatment with *cis* pT231-tau monoclonal mouse antibody (clone #113) or mouse IgG2b in a double-blind manner, as described^26,40^ with the following modifications. After rmdTBI, mice received intraperitoneal treatments (200 µg) on days 1, 7, 14 followed by once a week in 1.5 months, and twice a month for 2 months (with total 4 months of treatment) before analyses at 8 months. For all behavioral tests, experimenters were blinded to injury and treatment status, using color-coding stored in a password-protected computer.

### Spontaneous voiding assay

Group housed animals (4–6 per cage) were placed in a clean, empty cage lined with precut 3 MM acid-hardened filter paper (Waltham, MA) with one animal per cage. Voiding assays were conducted over 4 h per day for three consecutive days during which time mice had access to food but not water, as previously described^26,64^. Filter papers were imaged using UV light and analyzed using Image J Software using the threshold technique in double-blind manner. Image J particle analysis was performed on spots greater than 1.0 mm^2^ (corresponding to 1 μl urine), reducing non-specific marks potentially deposited by paws and tails that pass-through urine spots.

### Novelty Y-maze Test

Mice were longitudinally tested in Novelty Y-maze, which consisted of three closed arms in the Y-shape (50 cm.11 cm.10 cm) made of white plexiglas. This test includes two sessions. During training session, mice were pseudo-randomly assigned the two arms (the “start arm” and the “left or right arm”), allowing for a 3-min exploration of only these two arms of the maze. After a 1-min delay, Test session was started. During Test session, the mice allowed to explore freely all three arms of the maze for 3 min. Test session takes advantage of the innate tendency of mice to explore novel unexplored areas (e.g., the previously blocked arm). The time spent in novel unexplored areas of each animal was measured^79–81^. Mice first tested 2-months after the last injury, and then were retested 6-months later in the same maze but in a different environment. Mice with intact short-term memory prefer to explore a novel arm over the familiar arms, whereas mice with impaired spatial memory enter all arms randomly. Thus, test session represents a classic test for spatial working memory.

### Conscious cystometry

All instruments used were sterilized by autoclave. Mice were anesthetized with isoflurane (5% induction, 1.5% maintenance) from a precision vaporizer through tubing to a nose cone. The caudal ventral abdomen and back of the neck were clipped and prepped for sterile surgery. A midline incision (1–2 cm) was made in the abdomen immediately cranial to the pubis, and the bladder was exposed. A pursestring suture was positioned in the dome of the bladder with 8–0 suture, and a length of sterile polyurethane tubing (C30PU-RJV1307 Instech Labs, Plymouth Meeting, PA) was implanted into the bladder through an incision in the center of the pursestring suture. The suture was tightened and tied securing the catheter. The tubing was passed across the body wall and tunneled to the subcutaneous tissue of the back of the neck. The abdominal wall was closed with 5–0 absorbable suture. Skin wounds will be closed with 5–0 suture and Lidocaine gel (2%) liberally applied. The skin of the back of the neck was surgically prepared, and a small incision was made on the midline to exteriorize the catheter, which was then connected to a mouse vascular access harness (VAH) and the catheter is capped with a pinport (VAH and pinport from Instech Labs, Plymouth Meeting, PA) for aseptic infusion of fluids. The skin wound was closed with non-absorbable Ethicon silk sutures and Lidocaine gel applied. Animals were allowed to recover and given Meloxicam Sustained Release (SR) (4 mg/kg, SC) and enrofloxacin (5 mg/kg, SC) a single dose at surgery, to prevent potential infection and then returned to the animal care facility where they were housed individually until day of experiment, usually after 5–7 days after surgery. On the day cystometry was performed, the pinport was attached to the infusion and pressure recording system. The mouse is placed in a metabolic cage (Tecniplast, West Chester, PA) with filter paper (Cosmos blotting paper Legion Paper, New York, NY) and allowed to move freely during bladder filling through the catheter for about 1 hour of recording. Infusion was by syringe pump, model BS8000 (Braintree Scientific, Braintree, MA) and recording was via World Precision Instrument (Sarasota, FL) pressure transducer and TB4M amplifier, and via AD Instruments (Colarado Springs, CO) PowerLab analog-digital converter and associated LabChart software. Video recording was accomplished by placing an infra-red (IR) camera (FLIR C2, Nashua, NH) below the cage and a Logitech webcam (Freemont, CA) above the cage. Video recordings were made from both cameras to a personal computer running FLIRTools for IR recordings and recording software supplied with the Logiitech camera.

### Thermal video analysis

Mice were placed individually inside custom made 21 × 14 cm boxes with filter paper on the bottom. A FLIR model A65, (Nashua, NH) infrared thermal video camera was used to record 4 mice simultaneously from above for 2 hours. Recorded video was transferred to a computer and converted to Windows Media Video (WMV) format for viewing and analysis using FLIR ResearchIR software.

### Histology

#### Brain

Mice are euthanized via transcardial perfusion. They are deeply anesthetized with an overdose of chloral hydrate, 500 mg/kg, after which the heart is exposed via a thoracotomy. Following removal of the blood with phosphate buffered saline (20–30 ml), and after the heart had stopped beating, the animal was then perfused with 4% paraformaldehyde (20–30 ml) in the chemical fume hood. Brains were then removed for further analysis. Paraffin embedding and sectioning of the brain were carried out in the Harvard Rodent Histopathology Core.

#### Bladder

Whole bladders were excised from mice that had undergone pericardial infusion for brain recovery and immediately placed into 4% w/v phosphate buffered formaldehyde. Paraffin embedding, sectioning and H&E staining were carried out in the Beth Israel Deaconess Histology Core. Slides were imaged on an Olympus BX60 microscope (Center Valley, PA) and recorded with Olympus cellSens software.

### Immunostaining analysis

Immunostaining analysis was carried out as described^26,40^. The primary antibodies used were *cis* P-tau mAb (clone #25), hyperphosphorylated tau epitopes antibodies (AT8 and AT100) (Innogenetics), microglia-specific antibody (Iba1) (Wako), and neuonal nuclei specific antibody (NeuN) (Milipore). After treatment with 0.3% hydrogen peroxide, slides were briefly boiled in 10 mM sodium citrate, pH 6.0, for antigen enhancement. The sections were incubated with primary antibodies overnight at 4 °C. Then, biotin-conjugated secondary antibodies (Jackson ImmunoResearch) were used to enhance the signals. Manufacturer-supplied blocking buffer (Invitrogen) was used for each reaction. The sections were washed four times with TBS after each step. Labeled sections were visualized with a Zeiss confocal microscope. The gain of confocal laser was set at the level where there are no fluorescence signals including autofluorescence in sections without primary antibody but with secondary antibody (Fig. S10). Slides were imaged on a Leica SPE microscope (Leica Microsystems, Wetzlar, Germany) and VS120 slide scanner (Center Valley, PA). Immunostaining images and their co-localization were quantified using Fiji/ImageJ Coloc 2.

### Gallyas silver staining

For the detection of neurofibrillary-like tangles, a modified FD NeuroSilver staining methods (FD NeuroTechnologies) was used following the manufacturer’s instructions, as described^26,29,30^. The paraffin-embedded fixed tissue sections were deparaffinized and then dehydrated sequentially in 100%, 95%, and 70% ETOH for 5-min each, followed by washing in Milli-Q water 2 times, 5-min each. Then, we incubated sections first into a mixture containing equal volumes of Solutions A and B, 2 times, 10-min each, and then transferred them into a mixture consisting of equal volumes of Solution A and B with Solution E for 10-min according to the manufacturer’s instructions. In the next step, we placed the sections in a mixture of Solution C and F 2 times, 2-min each, and continued by incubating in a mixture of Solution D and F, followed by washing in Milli-Q water 2 times, 3-min each. For the final step, we placed the sections in Solution G at 4 °C for 1 hour. Sections were then covered with mounting media and cover slipped. As we know that a counterstain is used it should not mask the primary stain. Slides were imaged on a Keyence BZ X800 microscope (Keyence Corporation, Osaka, Japan). We did not use a counterstain such as the Nissl stain (cresyl violet) and Hematoxylin & Eosin (H&E) to avoid nuclear background labelling and masking effects that tend to occur with a counterstain along with Galyas Silver staining.

### Data acquisition and statistical analysis

We estimated the sample size considering the variation and mean of the samples. All surviving animals or samples were included in the analyses except a few mice died immediately after brain injury. Data acquisition and analysis obtained in an unbiased fashion. All data are presented as the means ± S.D. or S.E.M, followed by determining significant differences using the one-way ANOVA with Tukey post-hoc or two-way ANOVA with Bonferroni post-hoc tests, and significant p-values < 0.05 are shown.

### Blinding

Animals were randomly assigned groups for *in vivo* studies and for mAb treatment experiments, group allocation and outcome assessment were also done in a double-blinded manner. For all behavioral and histopathological tests, experimenters were blinded to injury and treatment status, using color-coding stored in a password-protected computer. Animals were also identified by ear tag and were kept as mixed populations of TBI + IgG, sham or TBI + *cis* mAb treated at 4–5 mice per cage. CMGs were performed and data analyzed prior to the specific treatment of each animal being revealed.

### Animals

All animal experiments were carried out in strict adherence with National Institutes of Health guidelines for animal care and use and with the approval of the Institutional Animal Care and Use Committee of Beth Israel Deaconess Medical Center.

## Supplementary information

Video-1

Video-2

Video-3

Supplementary Information
