# Supplementary material for: Traumatic Brain Injury-related voiding dysfunction in mice is caused by damage to rostral pathways, altering inputs to the reflex pathways
Source: Sci Rep. 2019 Jun 14;9:8646. doi: 10.1038/s41598-019-45234-8 (PMC6570649; doi:10.1038/s41598-019-45234-8)
Supplement: Supplementary file 4 — Supplementary Information [file 41598_2019_45234_MOESM4_ESM.pdf]

Traumatic Brain Injury-related voiding dysfunction in mice is caused by damage to rostral pathways, altering inputs to the reflex pathways

Onder Albayram*[1, 2, 3, 4], Bryce MacIver* [5], John Mathai[5], Anne Verstegen[5],

Sean Baxley[2, 3, 4], Chenxi Qiu[2, 3, 4, 6],Carter Bell[2, 3, 4], Barbara J. Caldarone[7], Xiao Zhen Zhou[2, 3, 4, 6]

Kun Ping Lu[2, 3, 4, 6], Mark Zeidel[5]

[1] Division of Cardiology, Department of Medicine,

Medical University of South Carolina, Charleston, SC 29425

[2] Hematology and Oncology Division, Department of Medicine,

Beth Israel Deaconess Medical Center, Harvard Medical School, Boston, MA 02215, USA.

[3] Division of Translational Therapeutics, Department of Medicine,

Beth Israel Deaconess Medical Center, Harvard Medical School, Boston, MA 02215, USA.

[4] Cancer Research Institute, Beth Israel Deaconess Medical Center,

Harvard Medical School, Boston, MA 02215, USA.

[5]Division of Nephrology, Department of Medicine

Beth Israel Deaconess Medical Center, Harvard Medical School, Boston, MA 02215, USA.

[6] Broad Institute of Harvard University and Massachusetts

Institute of Technology, Cambridge, MA 02142, USA.

[7]NeuroBehavior Laboratory, Harvard NeuroDiscovery Center, Harvard Medical School,

Boston, MA 02115, USA

* Equal contributions

Correspondence and requests for materials should be addressed to

O.A. (email: oalbayra@bidmc.harvard.edu or albayram@musc.edu) or

B.M. (email: imaciver@bidmc.harvard.edu)

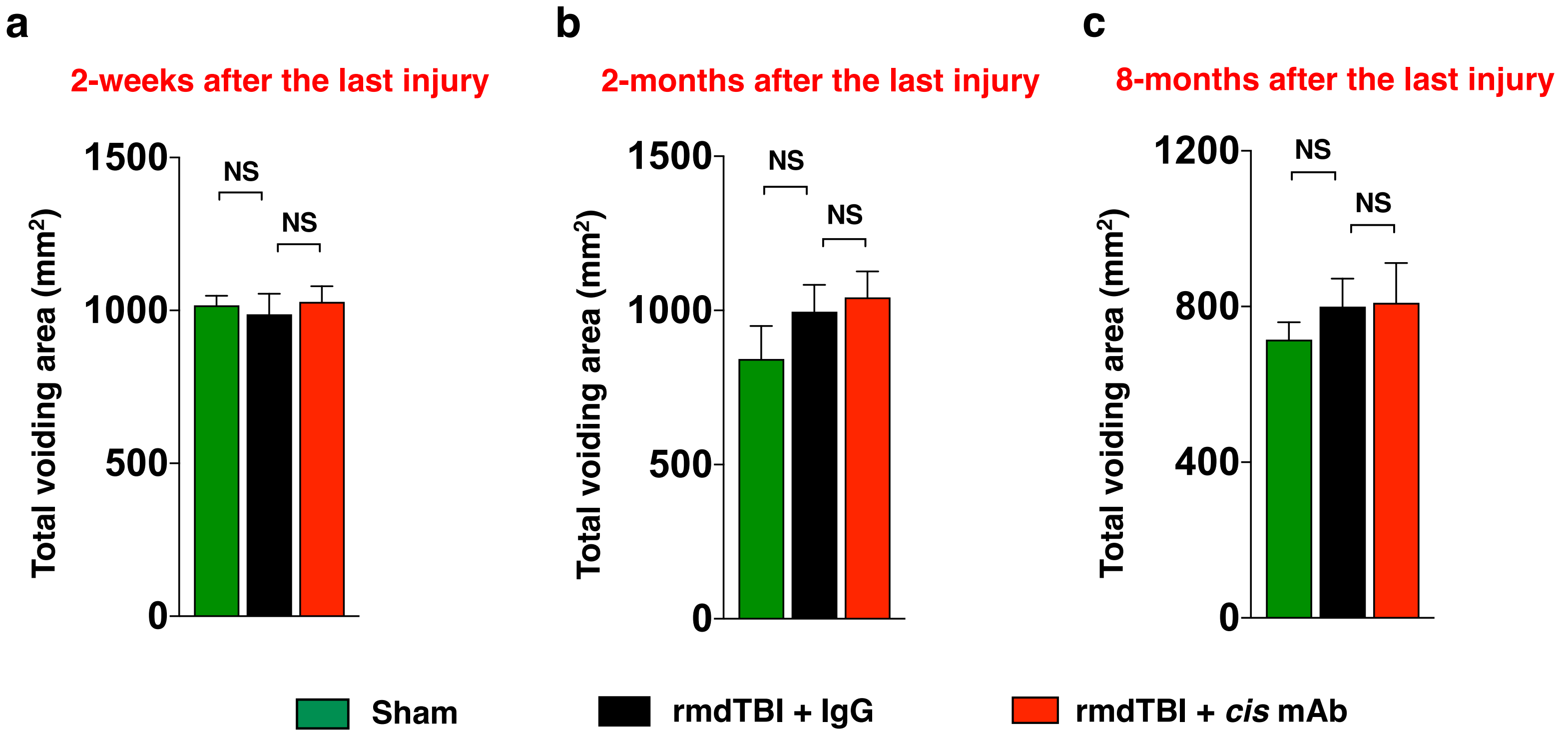

Supplementary Figure 1. There is no obvious total voiding area at 2 weeks, 2 months and 8 months after rmdTBI. Changes in total voiding area were analyzed using the spontaneous voiding assay at **(a)** 2-weeks, **(b)** 2-months, and **(c)** 8-months after the last injury. NS: not significant. N=4-5. The data were presented as means ± SEM. The p values were calculated using one-way ANOVA with post-hoc Tukey test.

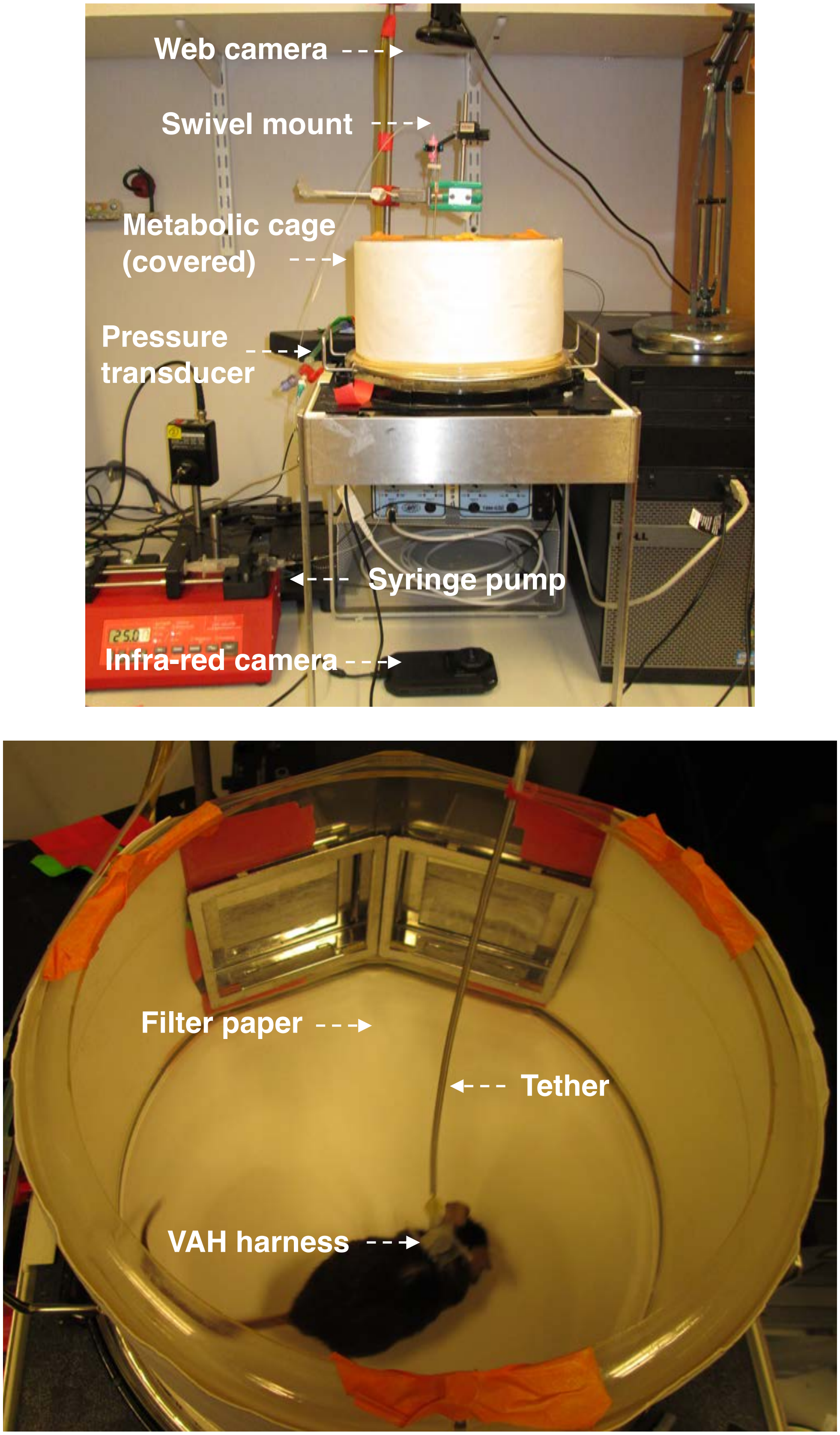

Supplementary Figure 2. Apparatus for performing Conscious Cystometry (CMG) allows detailed urodynamic assessment lower urinary track function in mice. Not pictured are data acquisition electronics.

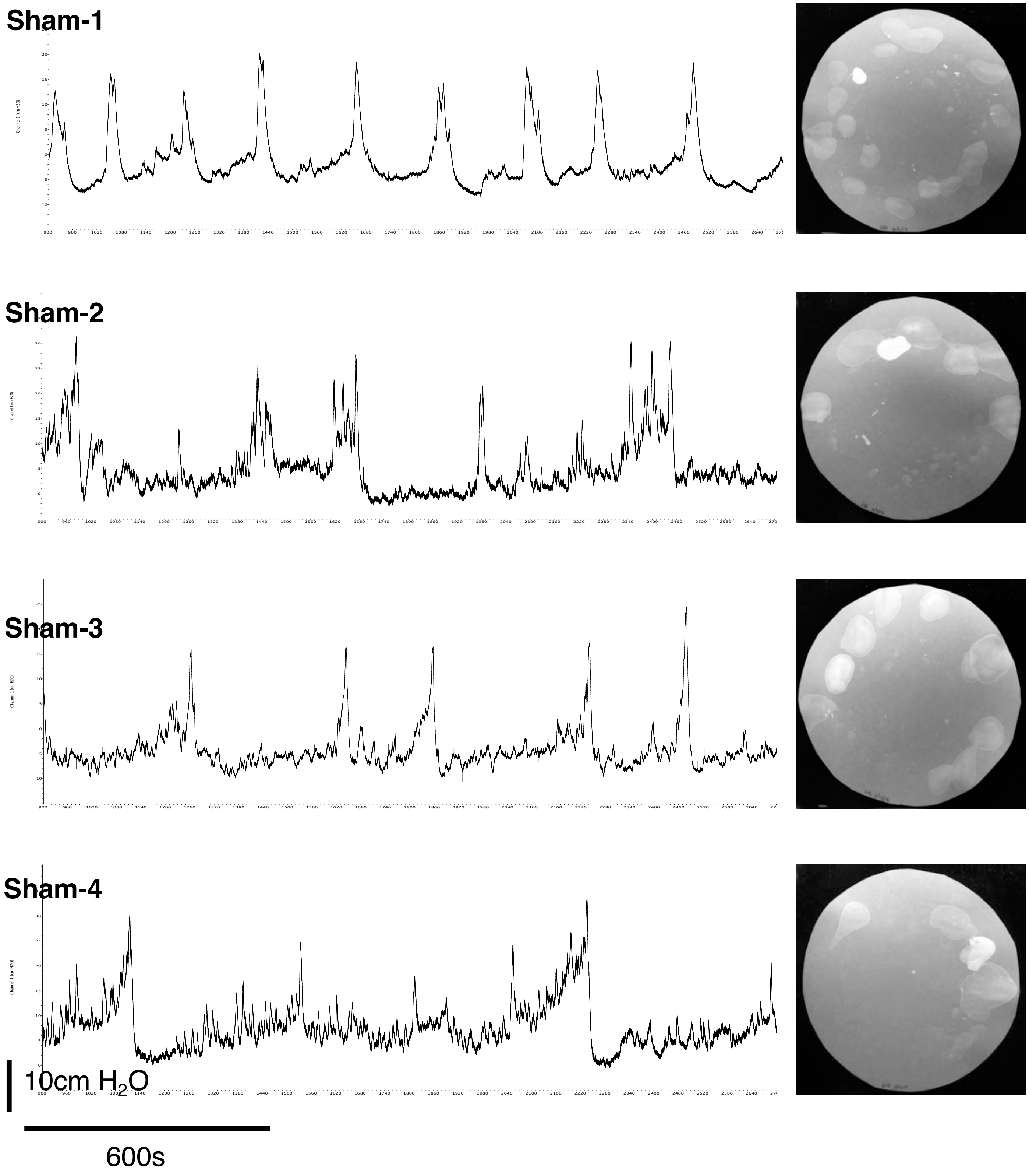

Supplementary Figure 3: . Urodynamic properties of sham mice tested at approximately 10 months of age. The second or third experiment for each animal is shown encompassing a 30 minute window from 900 to 2700s for the cystometrogram. The imaged final void spot filter is also presented.

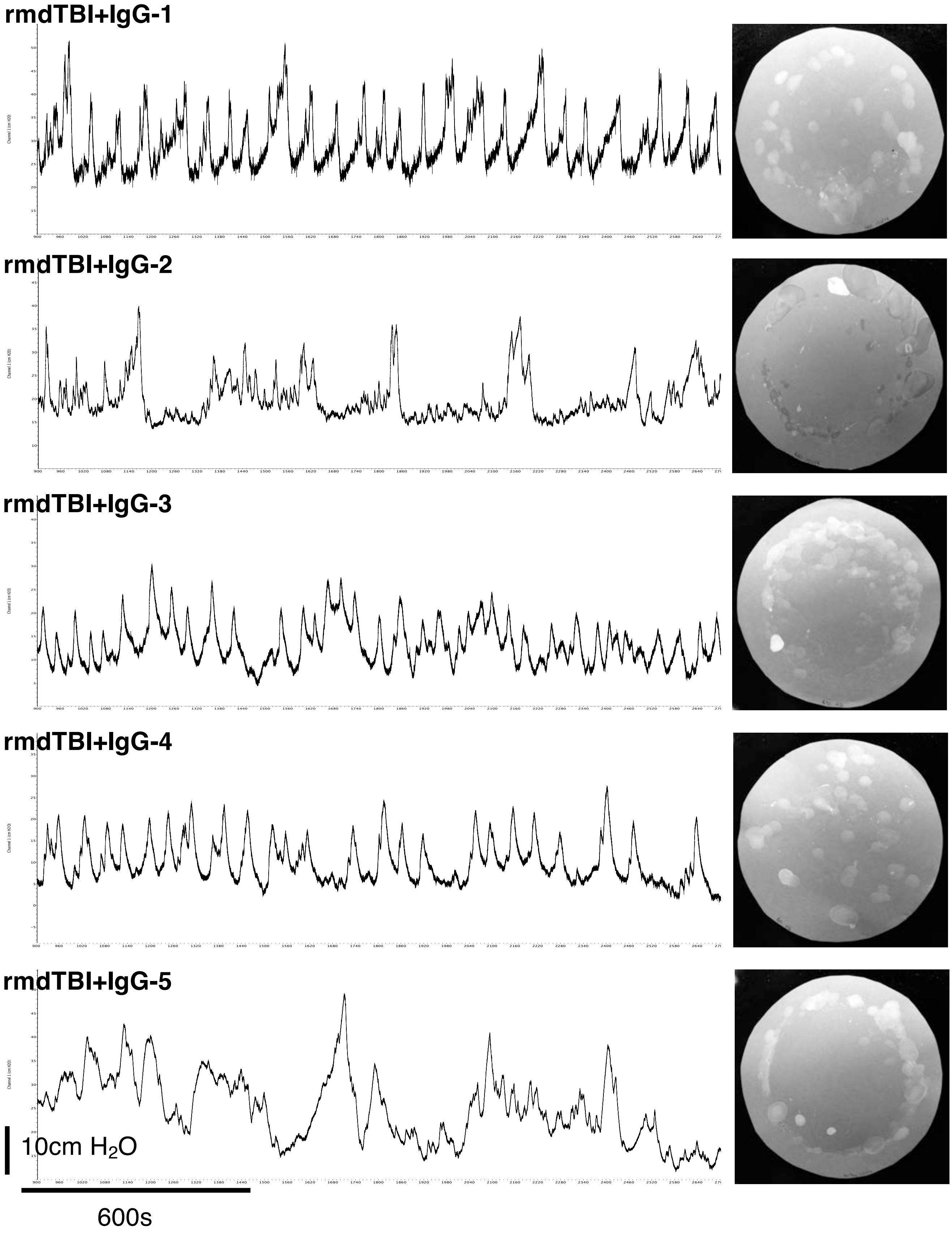

Supplementary Figure 4: . Urodynamic properties of rmdTBI mice treated with placebo IgG tested 8 months after the last injury at approximately 10 months of age. The second or third experiment for each animal is shown encompassing a 30 minute window from 900 to 2700s for the cystometrogram. The imaged final void spot filter is also presented.

rmdTBI+*cis* mAb-1

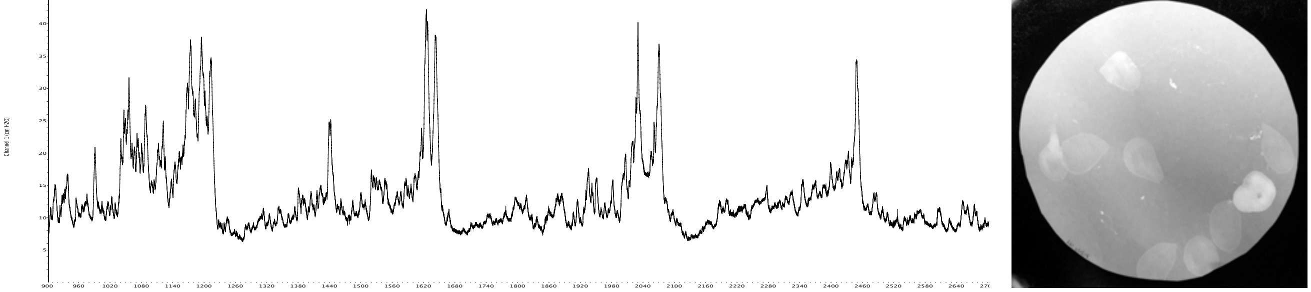

rmdTBI+*cis* mAb-2

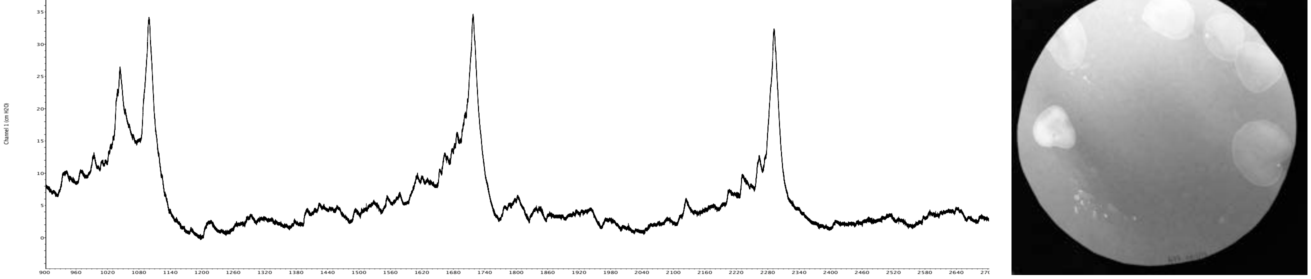

rmdTBI+*cis* mAb-3

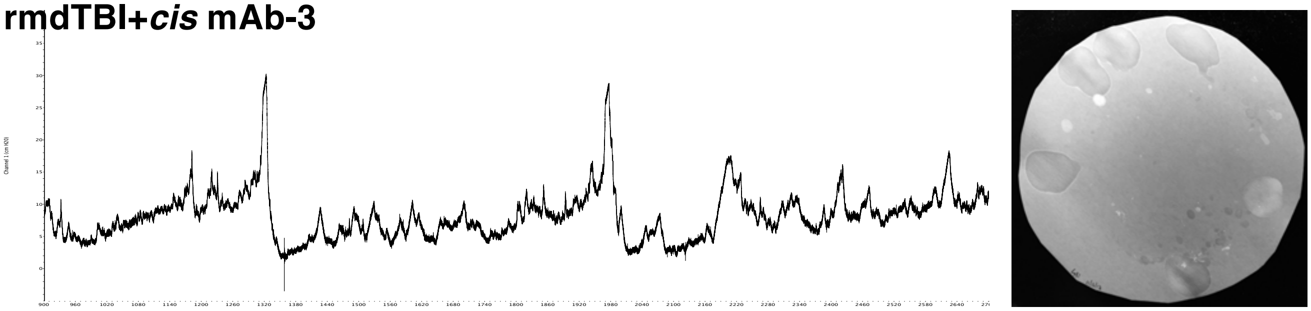

rmdTBI+*cis* mAb-4

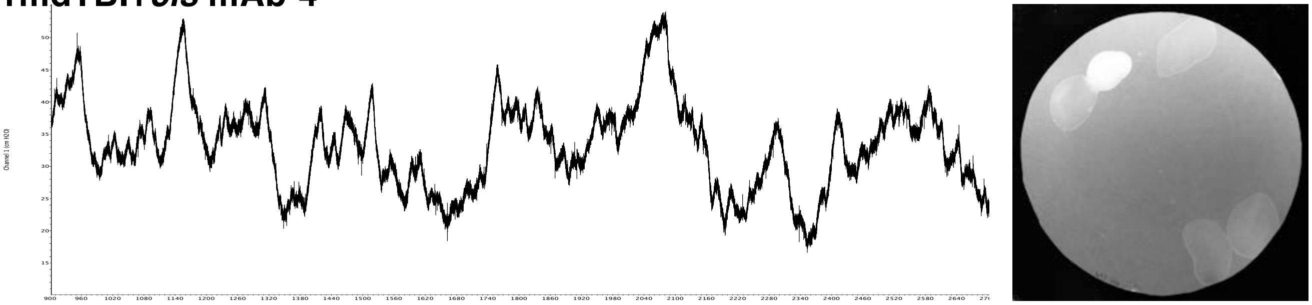

rmdTBI+*cis* mAb-5

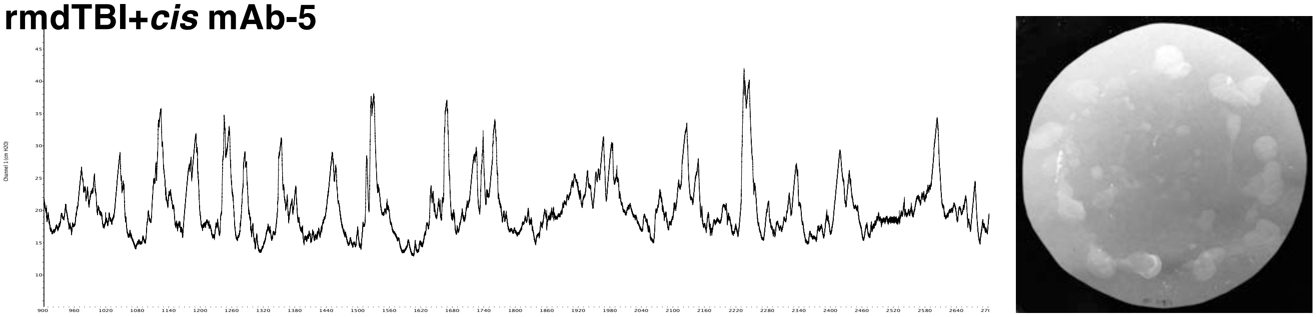

Supplementary Figure 5: . Urodynamic properties of rmdTBI mice treated with *cis* mAb tested 8 months after the last injury at approximately 10 months of age. The second or third experiment for each animal is shown encompassing a 30 minute window from 900 to 2700s for the cystometrogram. The imaged final void spot filter is also presented.

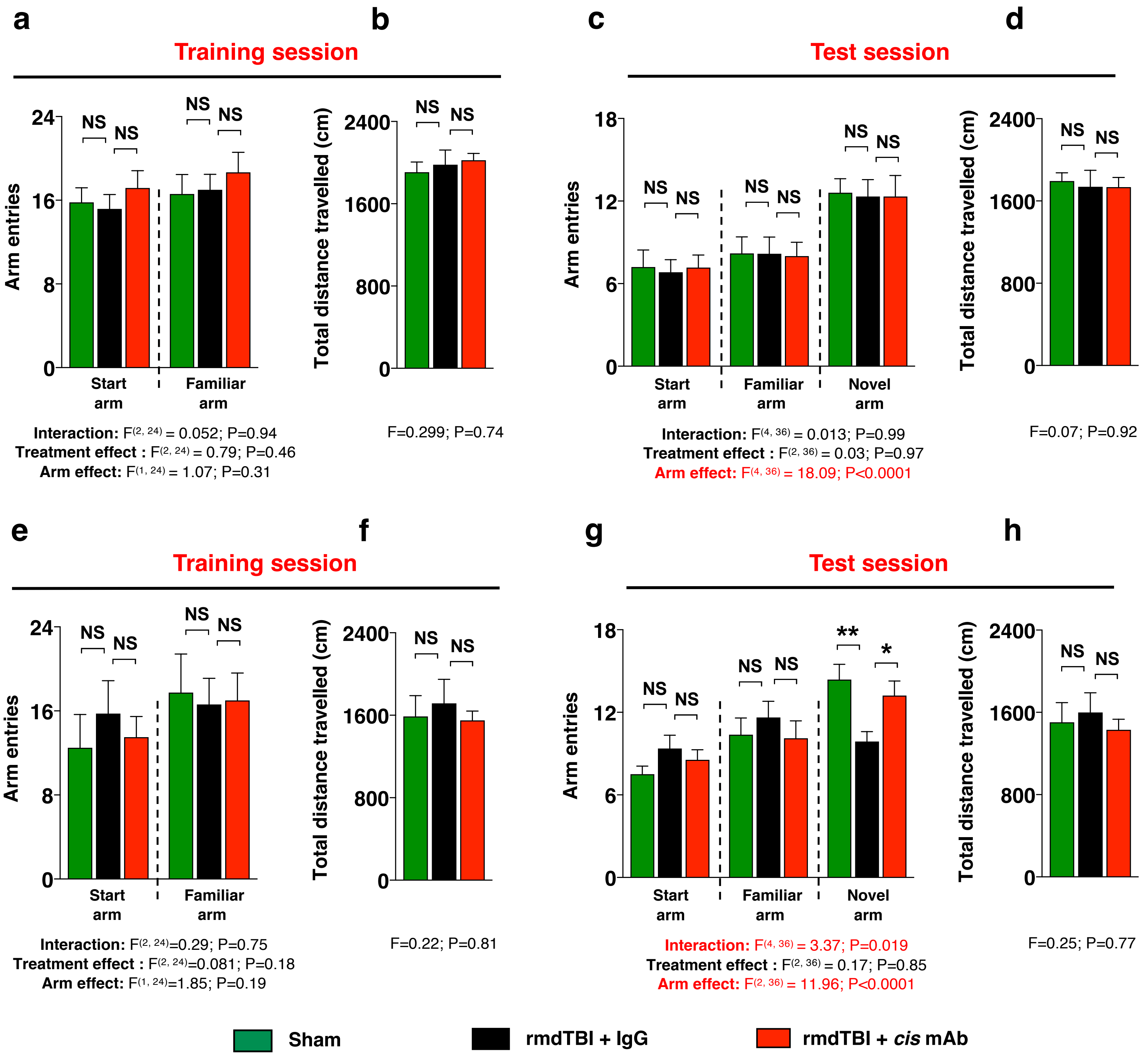

Supplementary Figure 6. There is no obvious difference in locomotor activity or anxiety-like behavior throughout tests at 2-and 8-months after injury. Number of arm entries in each training and test sessions at **(a, c)** 2-months, and **(e, g)** 8-months after the last injury. Total distance travelled in each training and test sessions at **(b, d)** 2-months, and **(f, h)** 8-months after the last injury. NS: not significant. The data were presented as means ± SEM. The p values were calculated using two-way ANOVA with post-hoc Bonferroni test or one-way ANOVA with post-hoc Tukey test *p<0.05, **p<0.01.

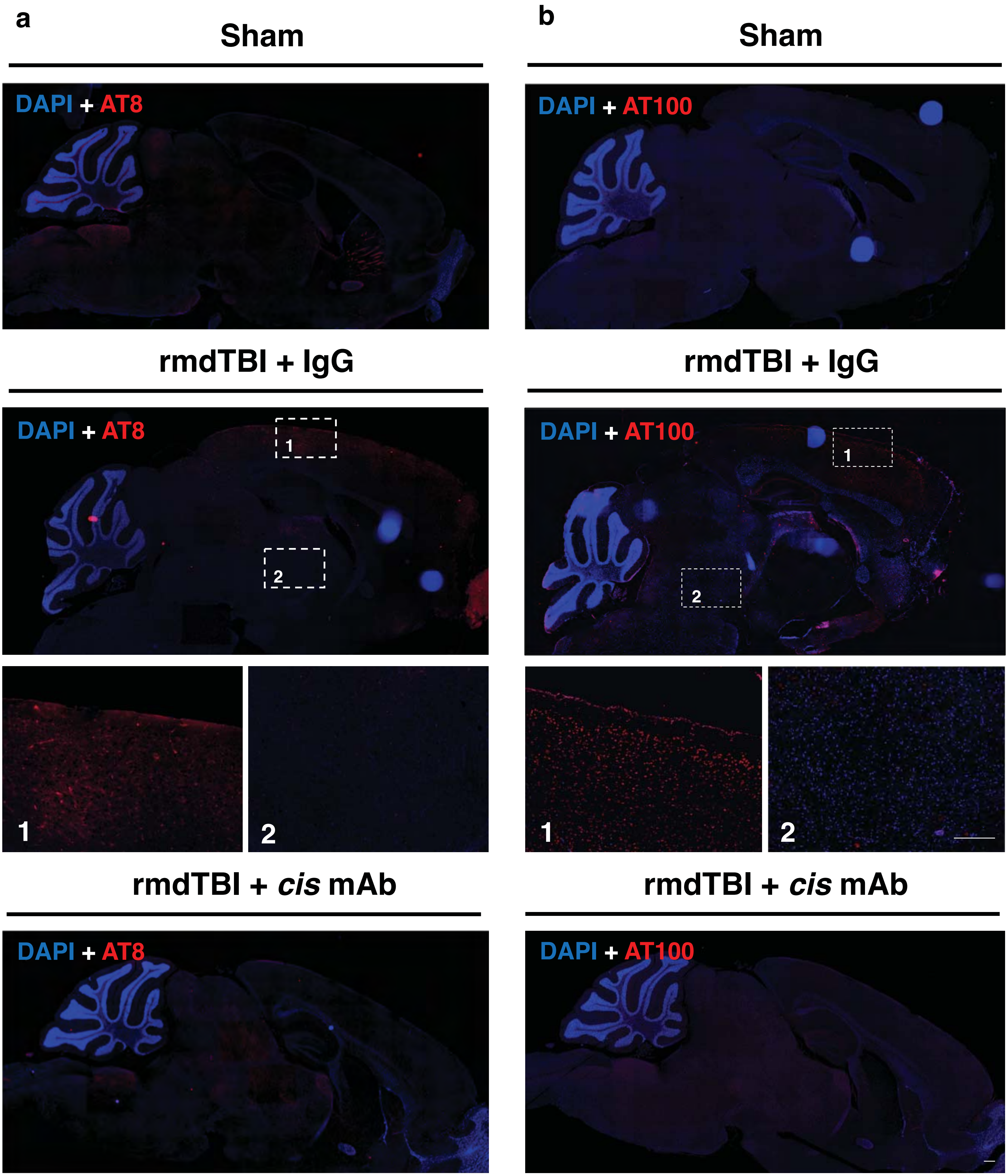

Supplementary Figure 7: There is a robust accumulation of tangle-like pathologies in cortex 8-months after repetitive moderate injury, but not in deeper brain regions. Sagittal brain sections of sham, rmdTBI+IgG, and rmdTBI+*cis* mAb mice were subjected to early-tangle marker AT8 **(a)** and late-tangle marker AT100 **(b)** along with DAPI at 8 months after injury. Lower panels are higher-magnification of the representative images comprised by the white dashed rectangle with corresponding numbers (1 and 2); Scale bar, 400 µm.

Gallyas silver staining

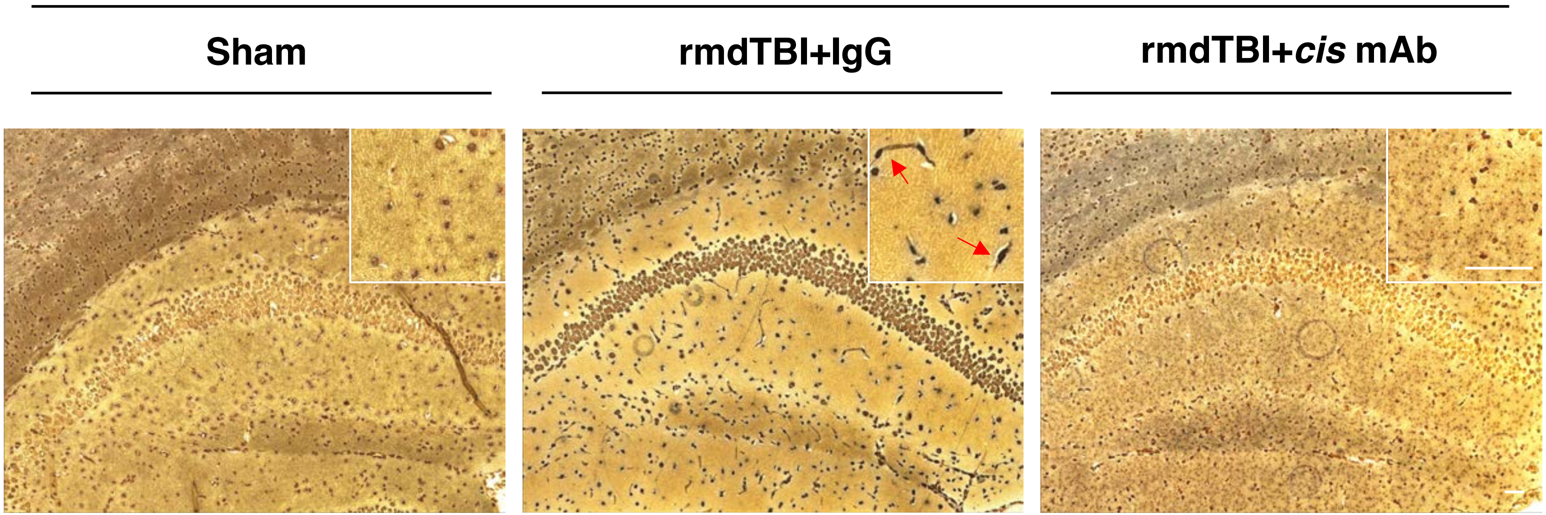

Supplementary Figure 7. Intracellular tangle-like inclusions were observed in hippocampal regions at 8 months after injury. Sagittal brain sections of sham, rmdTBI+IgG, and rmdTBI+*cis* mAb mice were subjected to Gallyas silver staining at 8 months after injury. Microscope images correspond to the hippocampal region. Inset images are high magnifications of representative Images. Red arrows point to tangle-like inclusions. Scale bar, 40 µm.

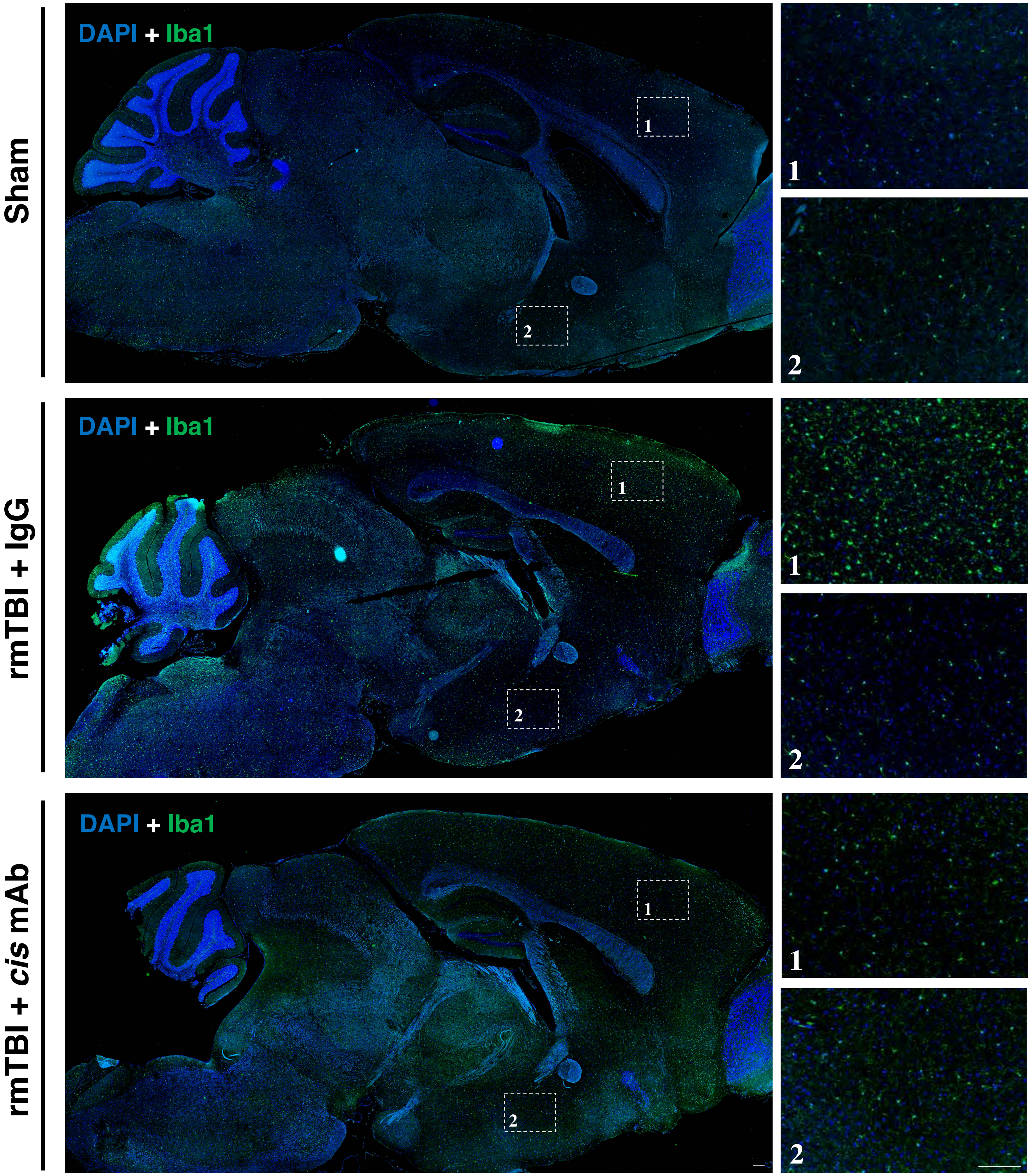

Supplementary Figure 9: Increased neuroinflammation in cortex, but not in deeper brain regions, 8-months after repetitive moderate injury that are closely correlated with a range of tau pathologies. Sagittal brain sections of sham, rmdTBI+IgG, and rmdTBI+*cis* mAb mice were subjected to microglia marker Iba1 along with DAPI at 8 months after injury. Inset images are high magnifications of representative areas with corresponding numbers (1 and 2); Scale bar, 400 µm.

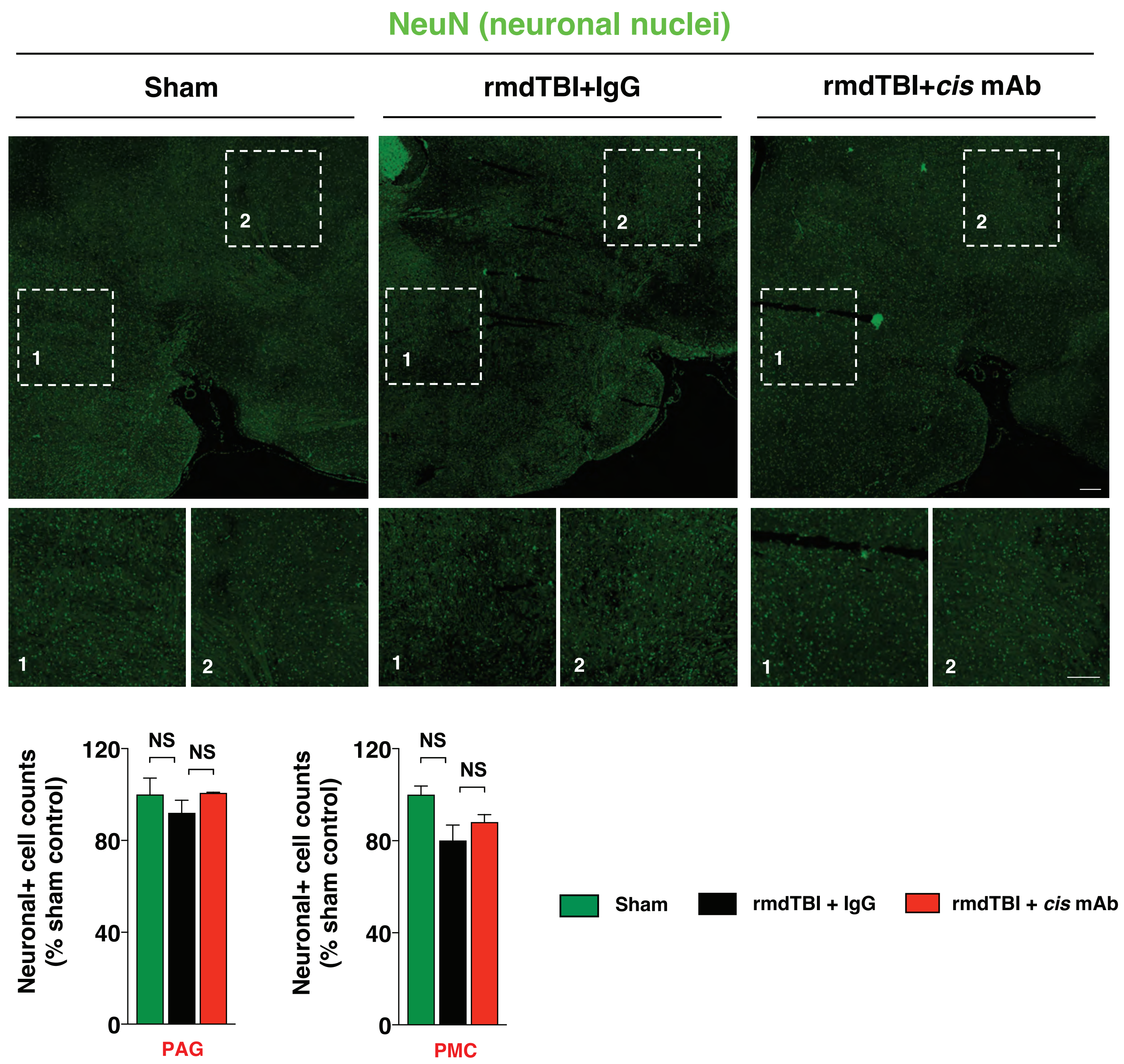

Supplementary Figure 10. No change was observed for neuronal density in PMC and PAG sub regions among the groups. Sagittal brain sections of sham, rmdTBI+IgG, and rmdTBI+*cis* mAb mice were subjected to neuronal nuclei marker, NeuN, at 8 months after injury. Inset images are high magnifications of representative brainstem-PMG and midbrain-PAG with corresponding numbers (1 and 2), respectively; Scale bar, 400 µm.The data were presented as means ± SEM of the percentage. NS: Not significant. The p values were calculated using one-way ANOVA with post-hoc Tukey test.

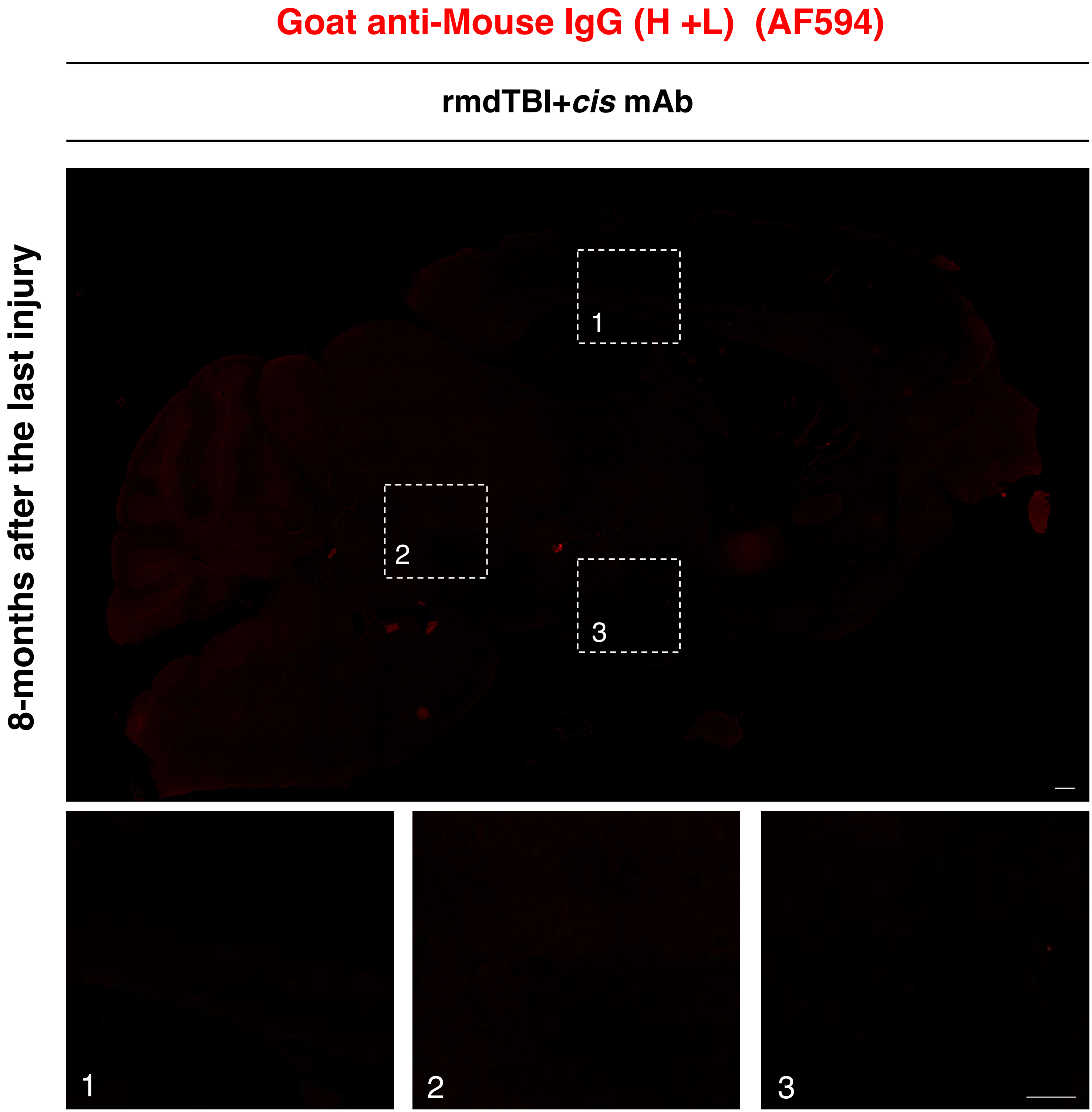

Supplementary Figure 11. Although we have four months wash-out period after the last antibody injection, we had indeed examined how the secondary antibody itself has provide signal in the brain. There was only a very slight autofluorescence, which were associated with white matter regions in whole brain section, however we did not observe any significant *cis* P-tau staining pattern. Scale bar, 400μm.
